# Age Estimation Based on CT Chest Analysis of Ossification of the Xiphisternal Joint in a Living Population Aged 35-50 in a Tertiary Setup

**DOI:** 10.7759/cureus.38160

**Published:** 2023-04-26

**Authors:** Padma V Badhe, Abhijeet D Shukla, Pauras Mhatre, Shashwat Shrivastava, Sanika Patil, Sanjay N Jain, Khushboo Tekriwal, Swapnil Moharkar, Moinuddin Sultan

**Affiliations:** 1 Radiology, Seth Gordhandas Sunderdas Medical College and King Edward VII Memorial Hospital, Mumbai, IND; 2 Radiology, Dr. Jankharia's Imaging Center, Mumbai, IND; 3 Radiology, Vedantaa Institute of Medical Sciences, Dahanu, IND

**Keywords:** chest ct, computed tomography, age estimation, ossification, xiphisternal joint

## Abstract

Introduction

Age estimation has been an area of special interest in the medicolegal context because of its necessity in various criminal and civil cases like assaults, murders, rapes, inheritance, insurance claims, etc. While legal documents are useful in daily activities that require age identity, they cannot be relied on for criminal and civil proceedings because of being falsifiable and inaccessible to some people. Scientific methods of age determination like physical, dental, and radiological examinations are used for reliable age estimation due to their universal and non-falsifiable nature. The skeletal examination is of great importance here because the human skeleton provides many sites for age estimation in different age groups. The xiphisternal joint between the xiphoid process and the body of the sternum provides one such opportunity in participants of 35-50 years of age. The ossification in this joint proceeds gradually in approximately the third to fifth decade of life; this natural variation in the morphology of the joint can be leveraged for age estimation. Previous studies showed that the mean age of fusion varied with the ethnicity of individuals and environmental factors. Thus, it is critical to have statistical information for the concerned population to avoid errors. Also, the relation of gender with the mean age of complete fusion remained ambiguous with the previous studies. The xiphisternal joint can be studied by radiological techniques like computed tomography (CT) and plain radiographs. Radiological methods have the benefit that they can be used on both living and dead participants and are non-invasive. The present study aims at gathering data relevant for use in India (Maharashtra) and to find out the reference age group in which there is complete ossification of the xiphisternal joint in males and females.

Methods and materials

This was a cross-sectional observational study in a tertiary care setup over a period of one year. High-resolution computed tomography (HRCT) was used for assessing joint fusion due to its high spatial resolution. The participants were included in the study if they were referred for HRCT chest by a physician for some pathology, did not have any trauma or lesion of the sternum and consented to the use of their information for the purpose of this study.

Results

The study included a total of 384 participants, out of whom 195 (50.8%) were males and 189 (49.2%) were females. The mean age of participants was 42.87 years. The mean age of complete xiphisternal joint fusion was observed to be 46.31 years (95% CI: 45.61 to 47.00) in males and 45.57 years (95% CI: 44.73 to 46.42) in females. Similarly, the mean age of participants with an unfused xiphisternal joint was observed to be 38.42 years (95% CI: 37.47 to 39.39) in males and 37.85 years (95% CI: 37.14 to 38.57) in females. There was no statistically significant difference in the age above which males and females show complete ossification of the xiphisternal joint.

Conclusion

The xiphisternal joint fusion can be used to determine the chronological age of an individual. It can be estimated as lesser than or equal to 45 years if the xiphisternal joint is unossified and greater than or equal to 37 years if the joint is ossified, with a 95% level of confidence.

## Introduction

Human identification is the determination of the individuality of a person based on certain physical characteristics [1]. Identification is necessary in various medico-legal scenarios like determining the age of criminal responsibility, statutory rape, victim trial by juvenile or adult court, and identification of victims in the case of decomposed remains. Age is a vital baseline parameter in both civil and criminal cases [2]. The legal age of consent, retirement age, concessions for senior citizens, and age of marriage are some of the important applications of age determination. Age determination from human skeletal remains for forensic and medico-legal purposes is an important matter in civil and criminal cases.

While legal documents like birth certificate, school mark sheets, voter IDs, etc. are good methods for age identification, they are not universal. Many undeveloped countries or regions do not have the infrastructure to register all citizens, due to which many people lack these documents, and we need to resort to alternate age-determination methods for them. Secondarily, such documents are falsifiable, many individuals fabricate their age in legal documents to gain advantages like fulfilling age thresholds for occupations, professional sports, and lawful marriage. In situations where age identity is critical, it is not correct to solely rely on the legal methods of age identification. Here, we need more accurate and universal scientific methods that cannot be falsified. These include physical examination, dental examination, and radiological/forensic post-mortem skeletal examination [3]. The skeletal examination makes use of multiple joints and bones of the body for age determination. The thoracic cage provides several options for age estimation. The medial end of the clavicles, manubriosternal joint, sternebrae, and xiphisternal joint, all have different age ranges of fusion/ossification and thus can be used for age estimation in various age groups.

Radiological methods have an advantage over other methods of skeletal analysis because they can be performed non-invasively and can be used in both living and dead subjects. Radiographs and computed tomography (CT) scans are two of the most viable options for skeletal imaging, within these radiographs suffer from the problem that the three-dimensional anatomy is superimposed on the two-dimensional radiographic plate. The quality of radiographic images thus formed has some restrictions like being non-linear and the poor scatter discrimination limits the ability to discern minute tissue variations [4]. Computed tomography (CT) is a technique for acquiring and reconstructing an image of a thin cross section using attenuation measurements. CT images, when compared with conventional radiographs, are free of the effects of superimposing tissues and can produce much higher contrast owing to a lack of scatter [4]. Thus, CT scans can be used for studying skeletal structure accurately and in high detail.

It has been observed in previous post-mortem and radiological studies that the xiphisternal joint remains unfused in the second decade of life, it starts to fuse in the third decade, and the fusion is completed by the fourth or fifth decades with some differences in males and females [2,5]. Thus, because of the natural age-related variation, the ossification status of the xiphisternal joint can be used to estimate the age of individuals. Previous studies on xiphisternal fusion showed that the statistical results vary greatly with ethnicity and environmental conditions. The results of previous studies were also affected by extreme values due to the wide age range of the sampled population. To make xiphisternal fusion usable in the forensic analysis as a reliable age estimation factor, we need to create population-specific standards. Despite a detailed literature review, there is a paucity of literature that investigates the ossification of the xiphisternal joint in living people in the Indian population, especially using radiological techniques. The present study aims at gathering data relevant for use in India (Maharashtra) and to find out the reference age group in which there is complete ossification of the xiphisternal joint in males and females and to explore the use of xiphisternal joint ossification as an age estimation parameter in the age group of 35 to 50 years.

## Materials and methods

The study was conducted across a period of one year, from December 2020 to December 2021, in a tertiary healthcare center in Maharashtra, India. This was a cross-sectional observational study in the age group of 35-50 years who fit in the inclusion criteria and needed CT chest for medical or surgical illness. The study was approved by the Institutional Ethics Committee (EC/89/2020) before beginning the study.

Patients who were referred for high-resolution computed tomography (HRCT) chest for some pathology, those who were in the age group 35 to 50 years and were Indian nationals by birth, were included in the study. Similarly, they were excluded from the study if they had any history of trauma or a pathological lesion of the sternum or had systemic bone diseases like osteoporosis. Written informed consent was obtained from the participants for inclusion in the study.

All the scans were performed on a 160-slice MDCT scanner, Toshiba Aquilion prime and Philips 64-slice Brilliance computed tomography unit. Information like age, sex, history of trauma to the sternum, and history of pathologies affecting bone structure was collected from the participants before performing the CT scan. The participants did not have to visit the tertiary care center solely for the purpose of this study. Before the CT scan, the patients were given instructions on how to hold their breath during the scan. A high-resolution CT chest scan was done of each subject. The scan covered a field of view covering the entire thorax from the lower neck till the upper abdomen using the following parameters: KV 120, effective mAs 250-790, time 0.35 s, detector collimation 0.625 mm, slice thickness 0.625-5 mm, matrix size 512 × 512. Tri-planar and three-dimensional evaluations of the xiphisternal joint were done on the CT console using 3D CT volume rendering technique (VRT), maximum intensity projection (MIP), and multiplanar reconstruction (MPR) projections with proper window settings.

**Figure 1:**
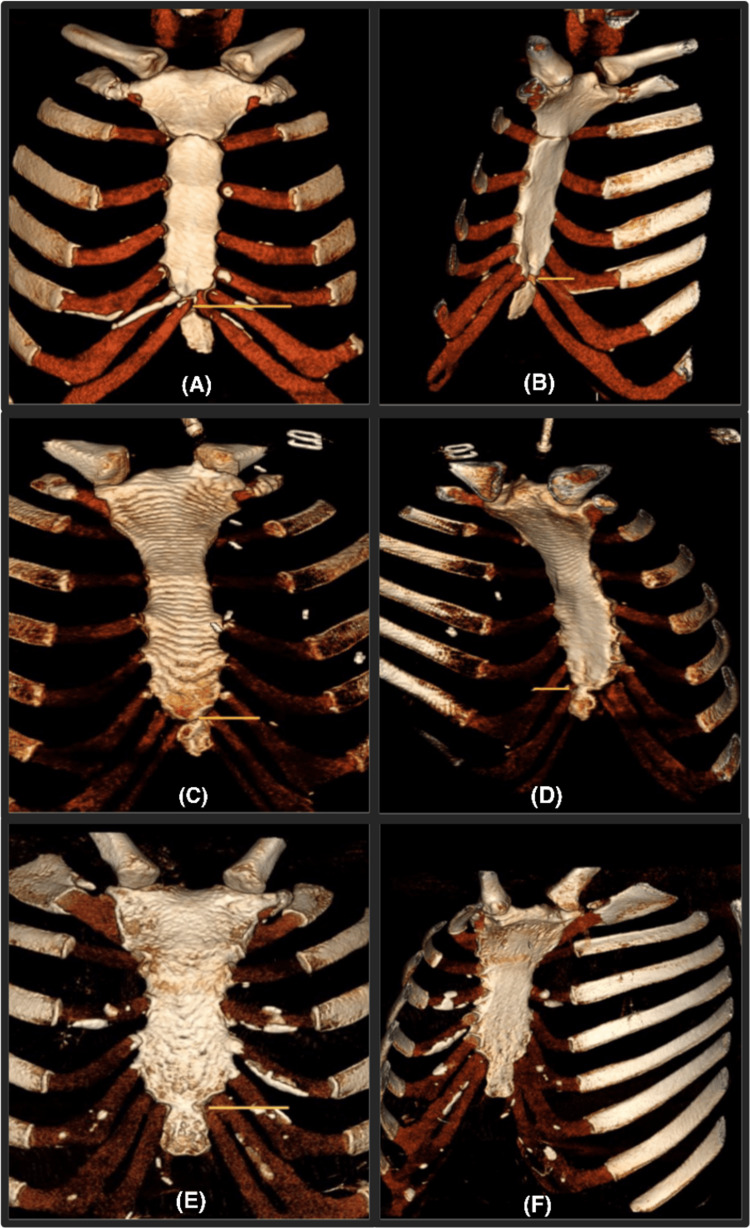
CT-3D volume rendered (VRT) image showing grades of xiphisternal joint ossification. (A) Unfused xiphisternal joint (grade 1-unfused joint), (B) posterior oblique view showing nonfusion at the joint, (C) partially fused xiphisternal joint (grade 2-partial fusion), (D) partial fusion of the xiphisternal joint more evident posteriorly, (E) completely fused xiphisternal joint (grade 3-complete fusion), and (F) posterior oblique view showing complete fusion of the joint. VRT: volume rendering technique.

According to Kaneriya et al. [6], the fusion of the xiphisternal joint can be graded as grade 1 for no fusion, grade 2 for partial fusion, and grade 3 for complete fusion (Figure 1).

The CT scans were examined according to this system analysis of the ossification status of the xiphisternal joint in our study. Participants were grouped into their respective categories based on the level of ossification in the xiphisternal joint.

Statistical analysis

Data were entered into Microsoft Excel (Windows 7; Version 2007, Microsoft Corporation, New York, USA), and analyses were done using the Statistical Package for Social Sciences (SPSS) for Windows Software (version 22.0; SPSS Inc., Chicago). Statistics such as mean and standard deviation (SD), frequencies and percentages were found out. Analysis was performed using the Fisher-Freeman-Halton exact test (for the association of fusion status between males and females), the independent samples t-test (for comparison of age at complete fusion between males and females), and Spearman’s rank correlation test (for correlation between age and percentage fusion status). p values less than 0.05 were considered statistically significant.

## Results

The study included a total of 384 participants, out of whom 195 (50.8%) were males and 189 (49.2%) were females. The age range of the study participants was 35 to 50 years of age. The mean age of the study participants was 42.87 ± 5.33 years. Table 1 shows the detailed age and gender distribution of participants in the study.

**Table 1:** Distribution of study participants by age and gender (N = 384).

Age (years)	Male (n (% of total N = 384))	Female (n (% of total N = 384))	Total (n (% of total N = 384))
35	14 (3.65%)	21 (5.47%)	35 (9.12%)
36	12 (3.13%)	19 (4.95%)	31 (8.08%)
37	10 (2.6%)	16 (4.17%)	26 (6.77%)
38	16 (4.17%)	13 (3.39%)	29 (7.56%)
39	4 (1.04%)	6 (1.56%)	10 (2.6%)
40	13 (3.39%)	10 (2.6%)	23 (5.99%)
41	6 (1.56%)	6 (1.56%)	12 (3.12%)
42	7 (1.82%)	6 (1.56%)	13 (3.38%)
43	8 (2.08%)	7 (1.82%)	15 (3.9%)
44	4 (1.04%)	10 (2.6%)	14 (3.64%)
45	16 (4.17%)	15 (3.91%)	31 (8.08%)
46	7 (1.82%)	6 (1.56%)	13 (3.38%)
47	14 (3.65%)	10 (2.6%)	24 (6.25%)
48	11 (2.86%)	11 (2.86%)	22 (5.72%)
49	23 (5.99%)	5 (1.3%)	28 (7.29%)
50	30 (7.81%)	28 (7.29%)	58 (15.1%)
Total	195 (50.8%)	189 (49.2%)	384 (100%)

**Figure 2:**
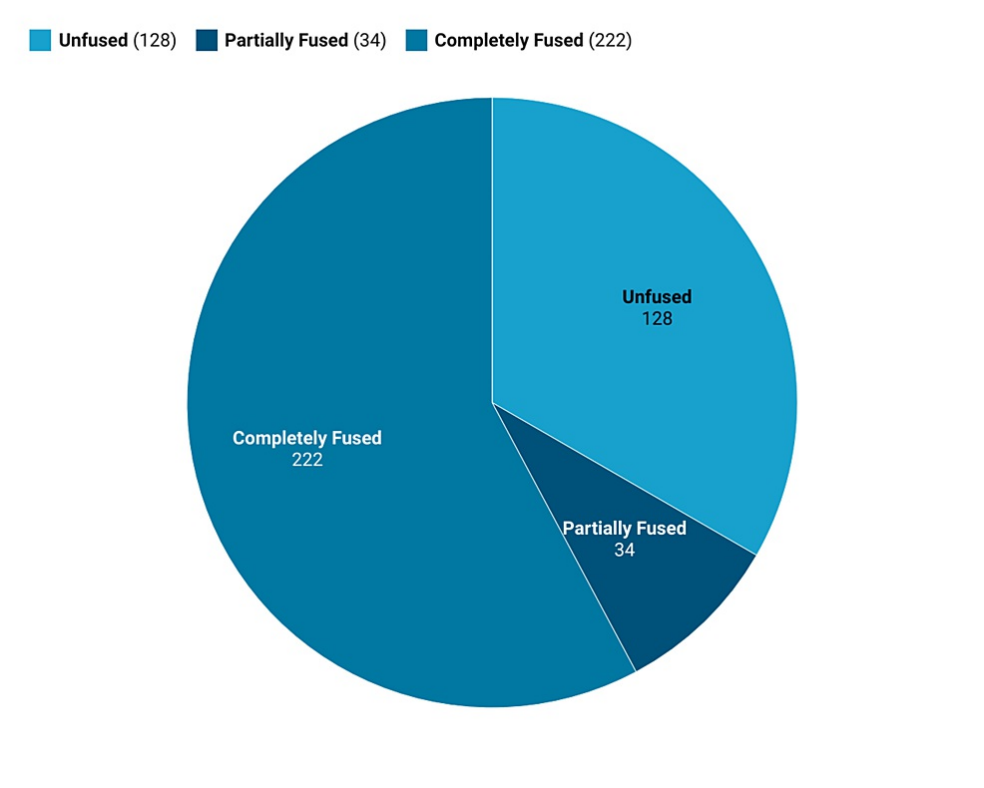
Distribution of study participants according to the status of fusion of the xiphisternal joint (N = 384).

As shown in Figure 2, out of all the participants (N = 384), 222 (57.8%) participants showed complete fusion of the xiphisternal joint in the form of complete ossification of the joint without any intervening cartilage in between. Thirty-four (8.9%) participants showed some degree of partial fusion (i.e., fusion had started but was not completed), and 128 (33.3%) showed a totally unfused joint with intervening cartilage and no ossification.

**Figure 3:**
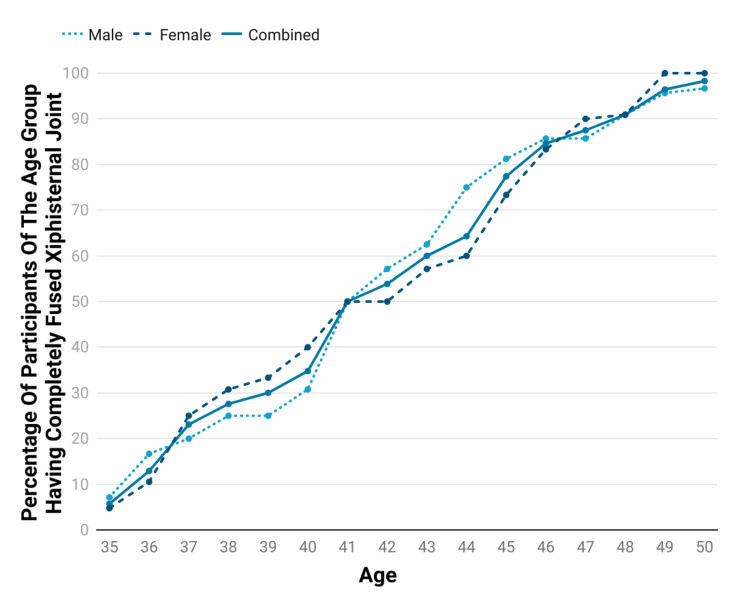
Graph showing the fusion status as a function of the age of the participants.

The percentage of participants with complete fusion of the xiphisternal joint varies directly with age (increasing as age increases). About 100% of the female participants and 96.6% of the male participants show complete fusion in the xiphisternal joint by the age of 50 years. Figure 3 shows the fusion status of the xiphisternal joint in females, males and combined as a function of the age of the participants. Spearman’s rho of the percentage of people with a completely fused xiphisternal joint correlated with age was calculated to be 0.999 for males, 0.999 for females, and 1.000 for both sexes combined (p-value less than 0.001 for all three correlations), thus showing a strong correlation of fusion status with age. 

For the same age, there was no significant effect of gender on the fusion status on the xiphisternal joint, as described in Table 2. The mean age of complete fusion of the xiphisternal joint was found to be 46.3 ± 3.9 years for males and 45.5 ± 4.3 years for females. This difference is statistically non-significant, with a p-value of 0.187 by t-test (t = 1.323). 

**Table 2:** Age-wise comparison of males and females for fusion status of the xiphisternal joint. *p values less than 0.05 are considered significant.

Age (years)	Sex	Status of fusion (n (% out of age-specific total))				Fischer-Freeman-Halton (exact) test*
		Unfused	Partially fused	Completely fused	Total	
35	Male	11 (78.57%)	2 (14.29%)	1 (7.14%)	14 (40%)	p >0.999
	Female	18 (85.71%)	2 (9.52%)	1 (4.76%)	21 (60%)	
	Total	29 (82.86%)	4 (11.43%)	2 (5.71%)	35 (100%)	
36	Male	10 (83.33%)	0 (0%)	2 (16.67%)	12 (38.71%)	p = 0.641
	Female	15 (78.95%)	2 (10.53%)	2 (10.53%)	19 (61.29%)	
	Total	25 (80.65%)	2 (6.45%)	4 (12.9%)	31 (100%)	
37	Male	5 (50%)	3 (30%)	2 (20%)	10 (38.46%)	p = 0.955
	Female	12 (75%)	0 (0%)	4 (25%)	16 (61.54%)	
	Total	17 (65.38%)	3 (11.54%)	6 (23.08%)	26 (100%)	
38	Male	9 (56.25%)	3 (18.75%)	4 (25%)	16 (55.17%)	p = 0.759
	Female	8 (61.54%)	1 (7.69%)	4 (30.77%)	13 (44.83%)	
	Total	17 (58.62%)	4 (13.79%)	8 (27.59%)	29 (100%)	
39	Male	3 (75%)	0 (0%)	1 (25%)	4 (40%)	p >0.999
	Female	3 (50%)	1 (16.67%)	2 (33.33%)	6 (60%)	
	Total	6 (60%)	1 (10%)	3 (30%)	10 (100%)	
40	Male	7 (53.85%)	2 (15.38%)	4 (30.77%)	13 (56.52%)	p >0.999
	Female	5 (50%)	1 (10%)	4 (40%)	10 (43.48%)	
	Total	12 (52.17%)	3 (13.04%)	8 (34.78%)	23 (100%)	
41	Male	1 (16.67%)	2 (33.33%)	3 (50%)	6 (50%)	p = 0.286
	Female	3 (50%)	0 (0%)	3 (50%)	6 (50%)	
	Total	4 (33.33%)	2 (16.67%)	6 (50%)	12 (100%)	
42	Male	0 (0%)	3 (42.86%)	4 (57.14%)	7 (53.85%)	p = 0.372
	Female	2 (33.33%)	1 (16.67%)	3 (50%)	6 (46.15%)	
	Total	2 (15.38%)	4 (30.77%)	7 (53.85%)	13 (100%)	
43	Male	3 (37.5%)	0 (0%)	5 (62.5%)	8 (53.33%)	p >0.999
	Female	2 (28.57%)	1 (14.29%)	4 (57.14%)	7 (46.67%)	
	Total	5 (33.33%)	1 (6.67%)	9 (60%)	15 (100%)	
44	Male	0 (0%)	1 (25%)	3 (75%)	4 (28.57%)	p >0.999
	Female	2 (20%)	2 (20%)	6 (60%)	10 (71.43%)	
	Total	2 (14.29%)	3 (21.43%)	9 (64.29%)	14 (100%)	
45	Male	1 (6.25%)	2 (12.5%)	13 (81.25%)	16 (51.61%)	p = 0.838
	Female	2 (13.33%)	2 (13.33%)	11 (73.33%)	15 (48.39%)	
	Total	3 (9.68%)	4 (12.9%)	24 (77.42%)	31 (100%)	
46	Male	1 (14.29%)	0 (0%)	6 (85.71%)	7 (53.85%)	p >0.999
	Female	0 (0%)	1 (16.67%)	5 (83.33%)	6 (46.15%)	
	Total	1 (7.69%)	1 (7.69%)	11 (84.62%)	13 (100%)	
47	Male	1 (7.14%)	1 (7.14%)	12 (85.71%)	14 (58.33%)	p >0.999
	Female	1 (10%)	0 (0%)	9 (90%)	10 (41.67%)	
	Total	2 (8.33%)	1 (4.17%)	21 (87.5%)	24 (100%)	
48	Male	1 (9.09%)	0 (0%)	10 (90.91%)	11 (50%)	p >0.999
	Female	1 (9.09%)	0 (0%)	10 (90.91%)	11 (50%)	
	Total	2 (9.09%)	0 (0%)	20 (90.91%)	22 (100%)	
49	Male	0 (0%)	1 (4.35%)	22 (95.65%)	23 (82.14%)	p >0.999
	Female	0 (0%)	0 (0%)	5 (100%)	5 (17.86%)	
	Total	0 (0%)	1 (3.57%)	27 (96.43%)	28 (100%)	
50	Male	1 (3.33%)	0 (0%)	29 (96.67%)	30 (51.72%)	p >0.999
	Female	0 (0%)	0 (0%)	28 (100%)	28 (48.28%)	
	Total	1 (1.72%)	0 (0%)	57 (98.28%)	58 (100%)	
Entire sample	Male	54 (27.69%)	20 (10.26%)	121 (62.05%)	195 (50.78%)	p = 0.055
	Female	74 (39.15%)	14 (7.41%)	101 (53.44%)	189 (49.22%)	
	Total	128 (33.33%)	34 (8.85%)	222 (57.81%)	384 (100%)	

Table 3 gives the summary statistics of age for different fusion statuses. The age after which 95% of the participants show complete fusion of the xiphisternal joint is 37 years (38 for males, 37 for females). On the other hand, it could be interpreted that the age after which the xiphisternal joints remain unfused in only 5% of the population is 45 years (46 for males, 44 for males). The difference between males and females is not significant.

**Table 3:** Summary statistics for age for different fusion statuses.

Fusion status	Unfused			Partially fused			Completely fused		
Sex	Male	Female	Combined	Male	Female	Combined	Male	Female	Combined
Total (number)	54	74	128	20	14	34	121	101	222
Range (min–max) (years)	35–50	35–48	35–50	35–49	35–46	35–49	35–50	35–50	35–50
Mean (years)	38.42	37.85	38.09	40.65	40.57	40.62	46.31	45.57	45.97
Standard deviation (years)	3.60	3.13	3.36	3.91	4.05	3.91	3.92	4.31	4.11
95% Confidence interval (years)	37.47–39.3 9	37.14–38.57	37.52–38.67	38.94–42.36	38.45–42.69	39.30–41.93	45.61–47.00	44.73–46.42	45.43–46.51
Age at fifth centile (years)	35.00	35.00	35.00	35.00	35.00	35.00	38.00	37.00	37.05
Age at 95th centile (years)	46.35	44.35	45.00	47.10	45.35	46.35	50.00	50.00	50.00

## Discussion

In the present study, the fusion status of the xiphisternal joint was defined using the classification system given by Kaneriya et al [6]. Various past studies have concluded that the xiphisternal joint remains unossified before 30 years of age [2]. Ossification starts in the third decade and is completed up to various degrees by 50 years of age [5]. In this study, our aim was to find out the mean age at which complete fusion of the xiphisternal joint takes place in males and females and to establish whether there is a significant difference between males and females.

Forensic age estimation is important in various legal scenarios, as discussed earlier. The help of radiologists is often sought by the police and other authorities for both medical and legal purposes. Various joints in the body are useful for the same, depending on the age range in question, and no single bone or joint can be used for the analysis of the entire age spectrum in human beings. The sternum is useful for age estimation because various parts of the sternum (sternebrae, xiphisternal joint, and manubriosternal joint) all fuse at different ages as has been found out in previous studies.

Our results are distinct from previous radiological studies based on both radiographs and CT scans. Alaa El-Din et al. [5] reported 100% xiphisternal joint fusion in both males and females in the age group of 60-65 with only 16% of males and 33% of females showing complete fusion by the age of 50 in a study conducted in the Egyptian population. Herath et al. [2] reported a mean age of fusion of 65.7 in males and 68.8 in females with a statistically significant difference between the mean ages of males and females in a study conducted in the Sri Lankan population. Monum et al. [7] reported the mean ages of complete fusion for males and females to be 68 and 72, respectively, in a study conducted in a Japanese population. The difference in results of these studies can be explained based on different ethnicity and environmental factors. Technical parameters such as the slice thickness of the CT scan can also affect the image resolution and thus influence the estimation of the age of complete ossification. Further, the intrinsic characteristics of the population sample can also affect the mean age.

In all the previously mentioned studies, the upper age limit of the participants was much beyond 50 years. The upper age limit in the Egyptian study is 60 years, and for the Japanese and Sri Lankan studies, it is beyond 80 years. Since the mean is affected by extreme values, the mean age of complete fusion in these studies is significantly higher than in our study. Our results are close to those reported by Umap et al. [8], who reported the age of complete fusion for females to be around 40 years and for males to be between 41 and 45 years. This can be explained based on ethnicity and environmental factors since this study was conducted in India in the same state of Maharashtra as the present study. This highlights the important point raised by Franklin et al. [3]: application of foreign standards for the estimation of biological attributes in human skeletons results in reduced accuracy. The degree of discrepancy between the results of a reference population and the population that is being studied is directly proportional to the degree of dissimilarity (increasing biological distance). Thus, the formation of population-specific standards is very important.

In contrast to the results of the study conducted by Herath et al. [2], who reported a significant difference in the mean age of complete fusion of the xiphisternal joint in males and females, our study found out that complete fusion was seen at a younger age in females than males; however, the difference was statistically insignificant.

There are some limitations to our study. Firstly, our lower age limit was 35, at which some participants showed complete fusion of the xiphisternal joint. Thus, fusion in some individuals must have started before 35 years of age, and the inclusion of these participants in the study sample would affect the overall mean age of fusion. The mean age is also affected by extreme values, and in our cases, there is a predominance of values at the extremes of ages, which would affect the mean age. Also, the socioeconomic and nutritional status of the participants was not taken into consideration. Nutritional status affects the growth and development of bones as well as the fusion of epiphyses. However, no previous studies have been conducted that evaluate the role of nutrition and socioeconomic status on the fusion of the xiphisternal joint, and thus future studies must be conducted to evaluate the same. We relied on hospital documents for assessing the chronological age of the participants. However, in a developing country, it is highly probable that the documented age differs from the real chronological age. Also, radiation exposure and a relatively high cost are disadvantages of using CT for age estimation. 

## Conclusions

We conclude that CT assessment of ossification of the xiphisternal joint is useful in the age estimation of living people. The mean age at which complete fusion occurs in males and females is 45-46 years. The chronological age of the individual can be estimated to be greater than or equal to 37 years when the xiphisternal joint shows complete fusion (with 95% confidence level). Similarly, in the case of an unossified xiphisternal joint, the age of the individual can be estimated as less than or equal to 45 years (with 95% confidence level). There is no significant difference in the age above which males and females show complete ossification of the xiphisternal joint.
